# The Readability and Quality of Web-Based Patient Information on Nasopharyngeal Carcinoma: Quantitative Content Analysis

**DOI:** 10.2196/47762

**Published:** 2023-11-27

**Authors:** Denise Jia Yun Tan, Tsz Ki Ko, Ka Siu Fan

**Affiliations:** 1 Department of Surgery Royal Stoke University Hospital Stoke on Trent United Kingdom; 2 Department of Surgery Royal Surrey County Hospital Guildford, Surrey United Kingdom

**Keywords:** nasopharyngeal cancer, internet information, readability, Journal of the American Medical Association, JAMA, DISCERN, artificial intelligence, AI

## Abstract

**Background:**

Nasopharyngeal carcinoma (NPC) is a rare disease that is strongly associated with exposure to the Epstein-Barr virus and is characterized by the formation of malignant cells in nasopharynx tissues. Early diagnosis of NPC is often difficult owing to the location of initial tumor sites and the nonspecificity of initial symptoms, resulting in a higher frequency of advanced-stage diagnoses and a poorer prognosis. Access to high-quality, readable information could improve the early detection of the disease and provide support to patients during disease management.

**Objective:**

This study aims to assess the quality and readability of publicly available web-based information in the English language about NPC, using the most popular search engines.

**Methods:**

Key terms relevant to NPC were searched across 3 of the most popular internet search engines: Google, Yahoo, and Bing. The top 25 results from each search engine were included in the analysis. Websites that contained text written in languages other than English, required paywall access, targeted medical professionals, or included nontext content were excluded. Readability for each website was assessed using the Flesch Reading Ease score and the Flesch-Kincaid grade level. Website quality was assessed using the Journal of the American Medical Association (JAMA) and DISCERN tools as well as the presence of a Health on the Net Foundation seal.

**Results:**

Overall, 57 suitable websites were included in this study; 26% (15/57) of the websites were academic. The mean JAMA and DISCERN scores of all websites were 2.80 (IQR 3) and 57.60 (IQR 19), respectively, with a median of 3 (IQR 2-4) and 61 (IQR 49-68), respectively. Health care industry websites (n=3) had the highest mean JAMA score of 4 (SD 0). Academic websites (15/57, 26%) had the highest mean DISCERN score of 77.5. The Health on the Net Foundation seal was present on only 1 website, which also achieved a JAMA score of 3 and a DISCERN score of 50. Significant differences were observed between the JAMA score of hospital websites and the scores of industry websites (*P*=.04), news service websites (*P*<.048), charity and nongovernmental organization websites (*P*=.03). Despite being a vital source for patients, general practitioner websites were found to have significantly lower JAMA scores compared with charity websites (*P*=.05). The overall mean readability scores reflected an average reading age of 14.3 (SD 1.1) years.

**Conclusions:**

The results of this study suggest an inconsistent and suboptimal quality of information related to NPC on the internet. On average, websites presented readability challenges, as written information about NPC was above the recommended reading level of sixth grade. As such, web-based information requires improvement in both quality and accessibility, and healthcare providers should be selective about information recommended to patients, ensuring they are reliable and readable.

## Introduction

### Background

The increase of technological advancements in the last 2 decades and equal access to digital devices has led to an increasing number of people in the United Kingdom with internet access [1-4]. This new digital age has changed the way health care information is accessed, with the internet becoming a valuable means for communicating health information to the general public [4-8]. Although patients with complex and chronic conditions can access information from a range of sources including medical journals and specialized support websites, the extent to which people can use and benefit from information on the internet often depends on the *health literacy* of the reader [1,4]. Health literacy, as defined by Berkman et al [2], is the ability to process, understand, and communicate health-related information for making informed decisions [1-4]. For individuals with low health literacy, evaluating and understanding health information may be more difficult, which may have negative consequences such as low adherence to medication or a misunderstanding of adverse effects [1,3,4].

The ever-evolving digital landscape and health care needs of the public have increased the need for more accessible information [5]. One key aspect of determining the accessibility of health care information on the internet is readability [6-8]. Readability is the estimation of the reading skills needed to comprehend a specific text to determine whether it can be understood by nonspecialist audiences such as patients [3,9]. This is typically measured in US school-grade levels [3,9]. The American Medical Association recommends that health care materials be written at or below a sixth-grade reading level [3,8]. Health information with poor readability may be inaccessible to individuals without medical training, which can have detrimental health consequences [6-8]. However, information that is easy to read may be beneficial to health and can therefore be a powerful resource for patients and health care professionals [1]. Websites such as NHS inform, Webmed, and Patient Info UK are written with a focus on readability, but many patients begin with an internet search, which can prioritize search engine optimization by pharmaceutical and other medical companies selling their products, with a higher risk of information bias.

Another significant barrier to the utility of health care information on the internet is quality [10,11]. Unlike traditional medical publishing media such as medical journals and textbooks, information on the internet is often not peer reviewed or edited, giving no guarantee of its accuracy [11,12]. Furthermore, no quality standard exists for health information websites, and individuals with low health literacy may have difficulty assessing the reliability or accuracy of the information [3,11-13]. Thus, this may result in inaccuracies and misinformation, and the quality of information reaching patients may be inconsistent [10,12,14].

### Objective

Nasopharyngeal carcinoma (NPC) is a rare cancer characterized by the formation of cancerous cells in the tissues of the nasopharynx. It is strongly associated with exposure to the Epstein-Barr virus (EBV), especially in its nonkeratinizing subtype, and has a higher incidence among those of Southeast Asian descent [15]. Although NPC is present worldwide, it is endemic in Asian and African countries [16]. The development of NPC involves a complex interplay between genetic susceptibility; environmental factors, such as dietary habits or the use of cigarettes; and EBV infection [15-19]. Typically, the treatment for NPC involves radiotherapy as the primary therapy, along with chemotherapy, adjuvant therapies, and surgical intervention for primary or recurrent lesions [16,17,20,21]. Diagnosis for most patients occurs in the mid to late disease stages when more obvious symptoms begin to appear and the cancer has begun to affect the surrounding organs [22]. This is because NPC clinically presents with nonspecific and variable symptoms during the initial stages. NPC can also remain clinically silent during the early stages owing to its deep location, with many patients being asymptomatic, thereby promoting disease progression [18,23,24]. The disease stage of NPC influences prognosis, with a 10-year survival at stage I of approximately 98% and a median survival of only 3 years in advanced stages, thereby emphasizing the importance of early detection [23,25]. Thus, despite many recent medical advancements, NPC is still responsible for approximately 50,000 deaths annually and approximately 800,000 new cases per year worldwide.

Although population screening strategies using methods such as EBV antibody assays have been shown to improve therapeutic outcomes, patients also play a crucial role in early diagnosis [20]. Individuals residing in endemic areas or patients with recurrent EBV infections must be particularly vigilant and informed enough to take the appropriate steps to limit risk factors and increase the chances of early detection [15,19,26].

Thus, access to high-quality, readable information is vital, particularly for patients, as it may improve early detection of the disease and provide support to patients during disease management. This study assesses the readability and quality of currently available web-based information about NPC to determine areas for improvement.

## Methods

### Search Strategy and Eligibility Criteria

In June 2023, the search terms “nasopharyngeal cancer,” “nasopharyngeal carcinoma,” “nasal cancer,” “nose cancer,” “sinus cancer,” “pharyngeal cancer,” and “throat cancer” were entered separately into the 3 most used internet search engines: Google, Bing, and Yahoo [27]. All cookies and browser history were cleared before the searches, and searches were performed in a private browsing window to minimize bias of results based on previous internet activity. The search was conducted in English, and websites were selected based on the order in which they appeared. The top 25 results generated for each term by each search engine were included, knowing that most internet users are unlikely to view results beyond that number. This generated 525 websites in total. From these, pages irrelevant to NPC, pages written in languages other than English, pages accessible only with paywall access or a subscription, pages targeting medical professionals such as articles presented in the format of a scientific paper, and pages containing content other than written information were excluded. Duplicated websites and websites that were subsections of other websites were also excluded. Websites were then grouped into categories independently by 2 authors (TK and DT), as shown in Multimedia Appendix 1.

Additional information, such as risk factors of NPC, red flag signs that suggest NPC, investigations, management options, safety netting advice, and any secondary prevention advice, was also recorded. Whether the websites included epidemiology of NPC, explanation of head and neck anatomy and physiology, explanation of etiology, mortality, and complication rates was also noted.

### Quality Assessment

The quality of written information provided by each website was assessed using 3 validated methods: the Health on the Net Foundation (HONcode) certification, the Journal of the American Medical Association (JAMA) benchmark criteria, and the DISCERN instrument.

The HONcode is a nonprofit organization that was founded in 1995 to promote transparent and reliable health information on the web. Although it is no longer updated and has been permanently discontinued on December 15, 2022, it has been the oldest and most valued quality marker for web-based health information. On the basis of strict standards, it provided HONcode certification only to websites that follow all 8 principles: authority, complementarity, confidentiality, attribution, justifiability, transparency, financial disclosure, and advertising policy. The HONcode seal certified a website for 1 year and was renewed yearly according to site compliance. Many studies have demonstrated that HONcode was associated with superior accuracy of medical information.

The JAMA benchmark criteria were established to determine the credibility, reasonability, and utility of medical information on the internet. Each website was graded as either 0 or 1 for the categories of “Authorship,” “Attribution,” “Disclosure,” and “Last updated” by evaluators, resulting in cumulative scores ranging from 0 to 4. A higher JAMA score is reflective of a higher-quality material. Further details of each JAMA criteria are presented in Textbox 1.

The DISCERN instrument, initially validated in 1999, is a 16-item tool used for assessing the quality and reliability of health care websites. It consists of 3 sections: section 1 (questions 1-8) assesses the reliability of a publication, whereas section 2 (questions 9-15) assesses how good the quality of information on treatment choices is, and section 3 (question 16) provides an overall rating of the literature quality. Each question is rated with a score of 1 to 5, and the ratings of all 16 questions are added up to provide the final score, with 80 being the highest and 16 being the lowest. A higher score implies a better quality and reliability of information. Scores are categorized as follows: very poor (16-29), poor (30-40), fair (41-51), good (52-63), and excellent (64-80). The specific questions are listed in Table 1.

**Description of the Journal of the American Medical Association (JAMA) benchmark criteria used to evaluate the credibility, reasonability, and utility of web-based medical information on nasopharyngeal carcinoma. Websites are graded as either 0 or 1 for each criterion and then summed to generate a total score. This tool is reproduced and distributed with permission from Zhang et al [28].**

- Authorship
  Authors and contributors, their affiliations, and relevant credentials should be provided.
- Attribution
  References and sources for all content should be listed clearly, and all relevant copyright information should be noted.
- Disclosure
  Website “ownership” should be prominently and fully disclosed, as should any sponsorship, advertising, underwriting, and commercial funding.
- Date updated
  Dates that content was posted and updated should be indicated.

**Table 1:** A description of the DISCERN scoring criteria used to evaluate the quality of web-based medical information on nasopharyngeal carcinoma. This tool is reproduced and distributed with permission from Raja and Fitzpatrick [29].

Section and question number		What is being investigated?	Question rating					
			No	Partially				Yes
**Section 1**								
	1	Are the aims clear?	1	2	3	4	5	
	2	Does it achieve its aims?	1	2	3	4	5	
	3	Is it relevant?	1	2	3	4	5	
	4	Is it clear what sources of information were used to compile the publication (other than the author or producer?)	1	2	3	4	5	
	5	Is it clear when the information used or reported in the publication was produced?	1	2	3	4	5	
	6	Is it balanced and unbiased?	1	2	3	4	5	
	7	Does it provide details of additional sources of support and information?	1	2	3	4	5	
	8	Does it refer to areas of uncertainty	1	2	3	4	5	
**Section 2**								
	9	Does it describe how each treatment works?	1	2	3	4	5	
	10	Does it describe the benefits of each treatment?	1	2	3	4	5	
	11	Does it describe the risks of each treatment?	1	2	3	4	5	
	12	Does it describe what would happen if no treatment is used?	1	2	3	4	5	
	13	Does it describe how the treatment choices affect overall quality of life?	1	2	3	4	5	
	14	Is it clear that there may be >1 possible treatment choice?	1	2	3	4	5	
	15	Does it provide support for shared decision-making?	1	2	3	4	5	
**Section 3**								
	16	On the basis of the answers to all these questions, rate the overall quality of the publication as a source of information about treatment choices	1	2	3	4	5	

### Readability Assessment

The readability of written information on each website was assessed using 4 validated readability assessment tools: the Flesch Reading Ease score (FRES), the Flesch-Kincaid grade level (FKGL), the Simple Measure of Gobbledygook (SMOG) index, and the Gunning-Fog Score (GFS) [3]. These mathematical formulas quantify the readability of written materials and differ in the weighting of individual factors such as the number of words per sentence and the number of syllables per word (Table 2) [3,30,31]. Multiple well-validated readability scores were used to improve results and to allow for a nuanced and balanced evaluation, as no single scoring system is recognized as the standard [3,30,31]. Written patient information for each website was transferred into an web-based calculator [32] and analyzed for readability [33].

FRES generates a score ranging from 0 to 100, with higher scores indicating content that is easier to read. Content with a score of 60 to 79 is classed as “average,” written at a suitable reading level for a 12- to 15-year-old, and a score of 80 to 100 is categorized as “easy to read,” deemed suitable for a 9- to 12-year-old. Content with a score of 0 to 60 is classed as “difficult” to read.

FKGL, GFS, and the SMOG index estimate the US academic grade level (number of years of education) necessary to comprehend written information. Both the GFS and the SMOG index assess the average sentence length and the number of “complex” words, defined as those consisting of ≥3 syllables. A sample of 10 sentences from the beginning, middle, and end of a body of text is used for both calculations.

**Table 2:** Description of the readability formulas used to evaluate the readability of web-based medical information on nasopharyngeal carcinoma and score interpretations. This table is reproduced and distributed with permission from Raja and Fitzpatrick [29].

Test name	Formula	Result range	Result interpretation
FRES^a^	206.835 – 1.015 × (words/sentences) – 84.6 × (syllables/words)	0-100	0-30: very difficult 30-50: difficult 50-60: fairly difficult 60-70: standard 70-80: fairly easy 80-90: easy 90-100: very easy
FKGL^b^	0.39 × (words/sentences) + 11.8 × (syllables/words) – 15.59	0-12	US grade level of education required to understand a text on the first reading
GFS^c^	0.4 × ([total words/total sentences] + 100 [complex words/total words])	0-20	6: sixth grade (United States), 7: seventh grade, 8: eighth grade, 9-12: high school, 13-17: college, and 17+: college graduate
SMOG^d^	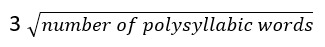	4-18	Years of formal education required to understand a text

^a^FRES: Flesch Reading Ease score.

^b^FKGL: Flesch-Kincaid grade level.

^c^GFS: Gunning-Fog Score.

^d^SMOG: Simple Measure of Gobbledygook.

Two authors (TK and DT) independently evaluated each website and were blinded to the others’ scoring, and the mean score was used in the analysis. This was done because both the JAMA benchmark criteria and the DISCERN instrument might impose a degree of bias on the person evaluating the website. Calculation of Cohen κ coefficient for interrater reliability was performed on the scores provided by each reviewer of the JAMA and DISCERN questionnaires. This was done to assess the degree of agreement between the 2 reviewers.

### Statistical Analysis

Statistical analyses were conducted using SPSS (version 25; IBM Corp) by KSF. Data were presented as mean for continuous variables and as counts with frequency percentages for categorical variables. ANOVA was used for the DISCERN score mean and JAMA score means comparison between web page groups. A 2-tailed *P* value of <.05 was used for statistical significance.

### Ethical Considerations

Ethics approval was not required for this study since all data was publicly available and there were no human subjects.

## Results

### Website Query and HONcode

**Figure 1:**
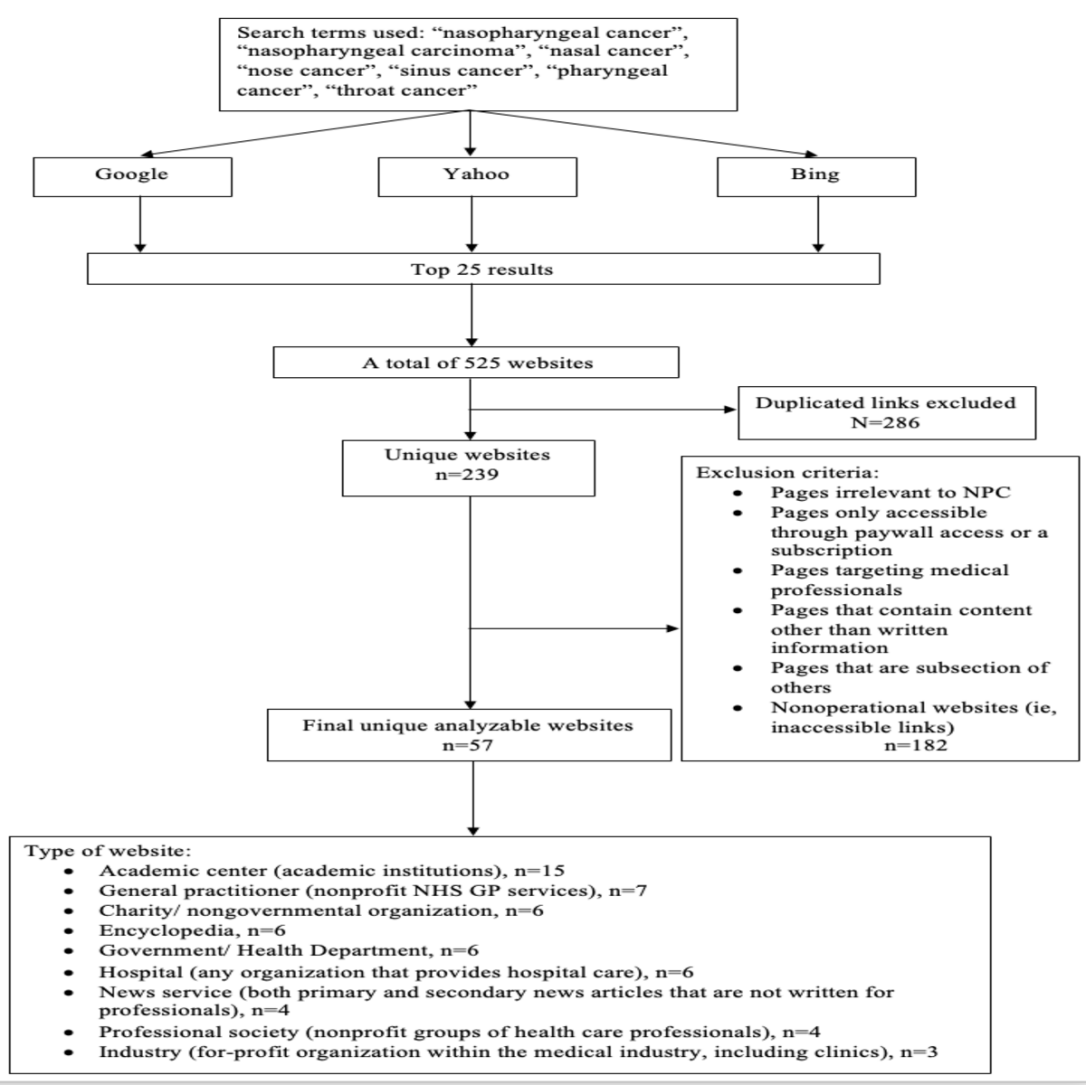
The PRISMA (Preferred Reporting Items for Systematic Reviews and Meta-Analyses) workflow for the identification of websites eligible for analysis. GP: general practitioner; NHS: National Health Service; NPC: nasopharyngeal carcinoma.

In total, 525 websites were identified. After the removal of intrasearch term duplicates and those that met the exclusion criteria, 57 websites remained for the analysis. Only 2% (1/57) of the websites within the study group had the HONcode seal present at the time of research, and it was the US-based encyclopedia website “ePainAssist,” which focuses on the prognosis of NPC [34]. The PRISMA (Preferred Reporting Items for Systematic Reviews and Meta-Analyses) workflow for the identification of websites eligible for analysis is shown in Figure 1.

### Quality Assessment

#### Journal of the American Medical Association

The overall mean JAMA score for all websites was 2.8 with a median of 3 (IQR 2-4; Table 3). In total, 60% (34/57) of the websites had a JAMA score >2. Overall, 33% (19/57) of the websites achieved the maximum score of 4, and 16% (9/57) of the websites achieved a score of 1. Industry websites (3/57, 5%) had the highest score of 4, followed by news services websites (4/57, 7%) and charity or nongovernmental organization websites (6/57, 11%) with a score of 3.8 and 3.7, respectively. In contrast, hospital websites (6/57, 11%) had the lowest score of 1.8, and general practitioner websites (7/57, 12%) had the second lowest score of 2. Significant differences were observed between the JAMA score of hospital websites and the scores of industry websites (*P*=.04), news service websites (*P*=.05), and charity or nongovernmental organization websites (*P*=.03). General practitioner websites were also found to have a significantly lower JAMA score than charity websites (*P*=.05). No significant differences were observed among the other website groups (Table S1 in Multimedia Appendix 2).

**Table 3:** Mean and median Journal of the American Medical Association benchmark criteria scores by different website source for nasopharyngeal cancer.

Website source	Score, mean (SD)	Score, median (IQR)
Industry	4 (0)	4 (4 -4)
News service	3.8 (0.5)	4 (3.75-4)
Charity or nongovernmental organization	3.7 (0.5)	4 (3.25-4)
Professional society	3.5 (1)	4 (3.5-4)
Encyclopedia	3.2 (0.4)	3 (3-3)
Government or health department	2.5 (0.8)	3 (2.25-3)
Academic center or institution	2.4 (1.4)	2 (1-4)
General practitioner	2 (0)	2 (2-2)
Hospital	1.8 (0.8)	2 (1.25-2)

In terms of country of origin, both China- (1/57, 2%) and US-based websites (31/57, 54%) had the highest JAMA score of 3, whereas Canada-based websites (1/57, 2%) had the lowest score of 2. No statistical difference in JAMA scores was observed between the websites of different countries (*P*=.36).

Multimedia Appendix 3 displays the number of websites that fulfilled the criteria for each category described by JAMA. The results revealed that “authorship” was fulfilled in 46% (26/57) of the websites, “attribution” in 54% (31/57) of the websites, “disclosure” in 100% (57/57) of the websites, and “date updated” in 77% (44/57) of the websites. None of the general practitioner websites and hospital websites scored for “authorship,” and none of the general practitioner websites scored for “attribution.” Only the HONcode-certified website scored a total JAMA score of 3, a result that was insignificant when compared with the average score of all other websites.

#### DISCERN

**Figure 2:**
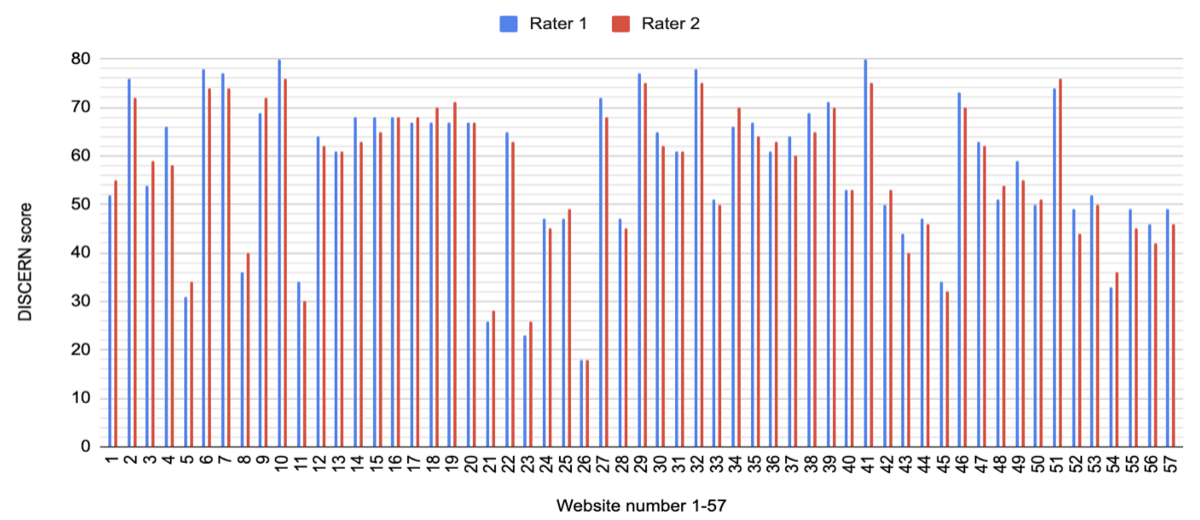
Overall DISCERN score for nasopharyngeal cancer.

The overall mean DISCERN score across all websites was 57.6 with a median score of 61 (IQR 49-68), a range of 18 to 80. The general DISCERN data properties are listed in Table 4, and the various scores are displayed in Figure 2. In total, 63% (36/57) of the websites had a DISCERN score of ≥52, a threshold that represents good-quality written materials. The 75th percentile of the total DISCERN score was ≥68, which was achieved by 28% (16/57) of the websites. These websites were considered high scoring.

More than half of all websites originated from the United States (31/57, 54%), with the third highest median total DISCERN score of 59. In total, 37% (21/57) of the websites from the United Kingdom were included, which had both the highest DISCERN score of 67 and the highest IQR of 21, followed by the Canada-based website (1/57, 2%) with a score of 66. China-based websites (1/57, 2%) had the lowest score of 31, and websites from Australia (3/57, 5%) had the second lowest score of 54. No statistical differences in DISCERN scores were found when comparing websites of different countries (Table 5).

Academic centers were the most common source of information (15/57, 26%) but had the second lowest median DISCERN score of 49 (Table 6). Charity or nongovernmental organization websites (6/57, 11%) had the highest DISCERN score of 77.5, followed by general practitioner 57ebsites (7/57, 12%), which scored 67, and new services websites (4/57, 7%), which scored 66.5. In contrast, websites with the lowest DISCERN score came from hospitals, with a score of only 36.5. General practitioner websites were found to have significantly higher total DISCERN scores than hospital websites (*P*=.03; Table S2 in Multimedia Appendix 2). The HONcode-certified website did not show a significantly greater DISCERN mean score when compared with websites without the HONcode seal. It had a total score of 50 only, which was lower than the mean of 57.6.

**Table 4:** DISCERN data properties of websites retrieved between June 2023 and July 2023 (N=57).

Overall DISCERN	Reliability	Quality	Total DISCERN, median
Values, median (IQR)	33 (25-36)	29 (21-33)	61 (49-68)
Lowest score	10	8	18
Highest score	40	40	80
99th percentile	40	40	80

**Table 5:** Descriptive analysis of the DISCERN quality assessment of nasopharyngeal carcinoma information on websites included in the quantitative analysis. Websites were retrieved between June 2023 and July 2023 and are categorized by country of origin (N=57).

Country	Articles, n (%)	Reliability score, median	Quality score, median	Total DISCERN, median (IQR)
Australia	3 (5)	30	26	54 (53-65)
Canada	1 (2)	34	32	66 (66-66)
China	1 (2)	15	16	31 (31-31)
United Kingdom	21 (37)	34	33	67 (47-68)
United States	31 (54)	33	28	59 (49-68)

**Table 6:** Descriptive analysis of websites included in the quantitative content analysis of web-based information on nasopharyngeal carcinoma retrieved between June 2023 and July 2023, categorized by source of information (N=57).

Website source	Articles, n (%)	Reliability score, median	Quality score, median	Total DISCERN, median (IQR)
Academic center (academic institutions)	15 (26)	28	28	49 (47-63)
Charity or nongovernmental organization (oversee a broader demographic, such as Red Cross and WHO^a^)	6 (11)	38	38.5	77.5 (46.25-78)
Encyclopedia	6 (11)	33.5	24.5	57 (50.25-63.75)
General practitioner (nonprofit)	7 (12)	34	33	67 (67-68)
Government or health department	6 (11)	31.5	25	55.5 (51.25-70.25)
Hospital (any organization that provides hospital care)	6 (11)	18.5	18	36.5 (23.75-57.5)
Industry (for-profit organization within the medical industry, including clinics)	3 (5)	34	29	66 (63.5-66.5)
News service (both primary and secondary news articles that are not written for professionals)	4 (7)	36	30	66.5 (55.75-69)
Professional society (nonprofit groups of health care professionals)	4 (7)	35.5	29.5	62.5 (53.75-73.25)

^a^WHO: World Health Organization.

The overall reliability of the websites was determined by section 1 (questions 1-8) of the DISCERN instrument, and the overall quality was determined by both sections 2 and 3 (questions 9-16). Although there was no statistical difference in the overall quality score between any website (*P*=.06), significant differences in the overall reliability scores were observed between websites of different sources. Charity or nongovernmental organization websites had the highest reliability scores (38), followed by news service websites (36) and professional society websites (35.5). Hospital websites had the lowest reliability score of 18.5, which was significantly lower than charity websites (*P*=.01), industry websites (*P*=.05), professional society websites (*P*=.03), and general practitioner websites (*P*=.008; Table S2 in Multimedia Appendix 2).

### Interrater Reliability

The level of agreement for each category for the JAMA benchmark criteria ranged from κ=0.71 (95% CI 0.33-1.10) for “date updated” to κ=0.93 (95% CI 0.83-1.03) for “authorship.” All κ coefficient values for the JAMA scores achieved an “almost perfect” rating (Table 7). For the DISCERN scores, the level of agreement for each question ranged from κ=0.21 (95% CI −0.05 to 0.48) for question 1 to κ=0.87 for question 13. In total, 7 out of 16 DISCERN items received a “substantial” level of agreement between the 2 raters, with 2 achieving an “almost perfect” agreement, 6 receiving “moderate” agreement, and 1 receiving “fair” agreement (Table 8).

**Table 7:** Summary of the interrater agreement for Journal of the American Medical Association (JAMA) quality assessment scores obtained for web-based information on nasopharyngeal carcinoma retrieved between June 2023 and July 2023 (N=57).

JAMA item	Cohen κ coefficient (95% CI)	Level of agreement
Authorship	0.93 (0.83-1.03)	Almost perfect
Attribution	0.89 (0.78-1.01)	Almost perfect
Disclosure	0.84 (0.53-1.15)	Almost perfect
Date updated	0.71 (0.33-1.10)	Substantial

**Table 8:** Summary of the interrater agreement for the DISCERN quality assessment scores obtained for web-based information on nasopharyngeal carcinoma retrieved between June 2023 and July 2023 (N=57).

DISCERN Item	Cohen κ coefficient (95% CI)	Level of agreement
1	0.21 (−0.05 to 0.48)	Fair
2	0.45 (0.24 to 0.66)	Moderate
3	0.49 (0.29 to 0.68)	Moderate
4	0.61 (0.42 to 0.79)	Substantial
5	0.63 (0.43 to 0.83)	Substantial
6	0.59 (0.41 to 0.76)	Moderate
7	0.49 (0.33 to 0.65)	Moderate
8	0.65 (0.49 to 0.80)	Substantial
9	0.86 (0.72 to 0.99)	Almost perfect
10	0.55 (0.38 to 0.73)	Moderate
11	0.72 (0.57 to 0.88)	Substantial
12	0.61 (0.46 to 0.77)	Substantial
13	0.87 (0.76 to 0.98)	Almost perfect
14	0.47 (0.22 to 0.73)	Moderate
15	0.6 (0.45 to 0.75)	Substantial
16	0.65 (0.49 to 0.81)	Substantial

### Readability

**Figure 3:**
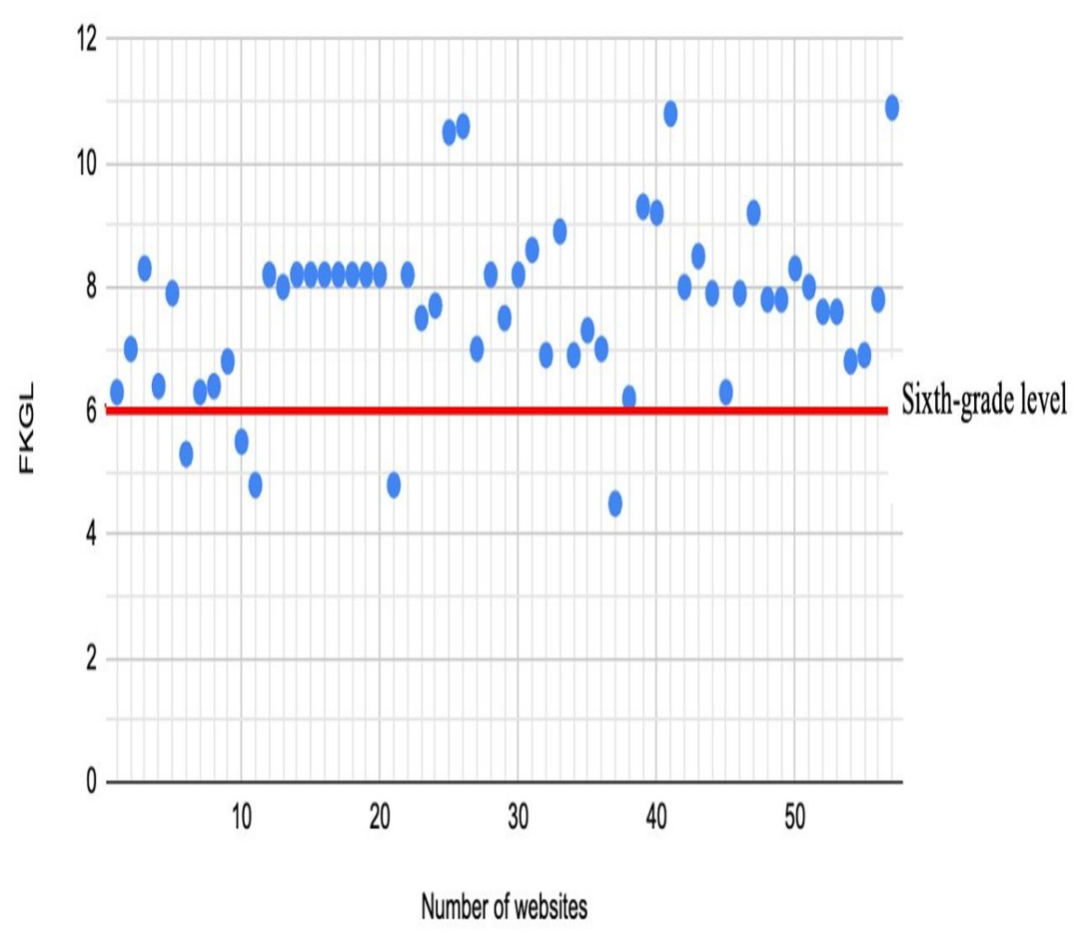
Scatterplot analysis of Flesch-Kincaid grade level (FKGL) scores; only 5 websites were at or below the recommended reading level.

**Figure 4:**
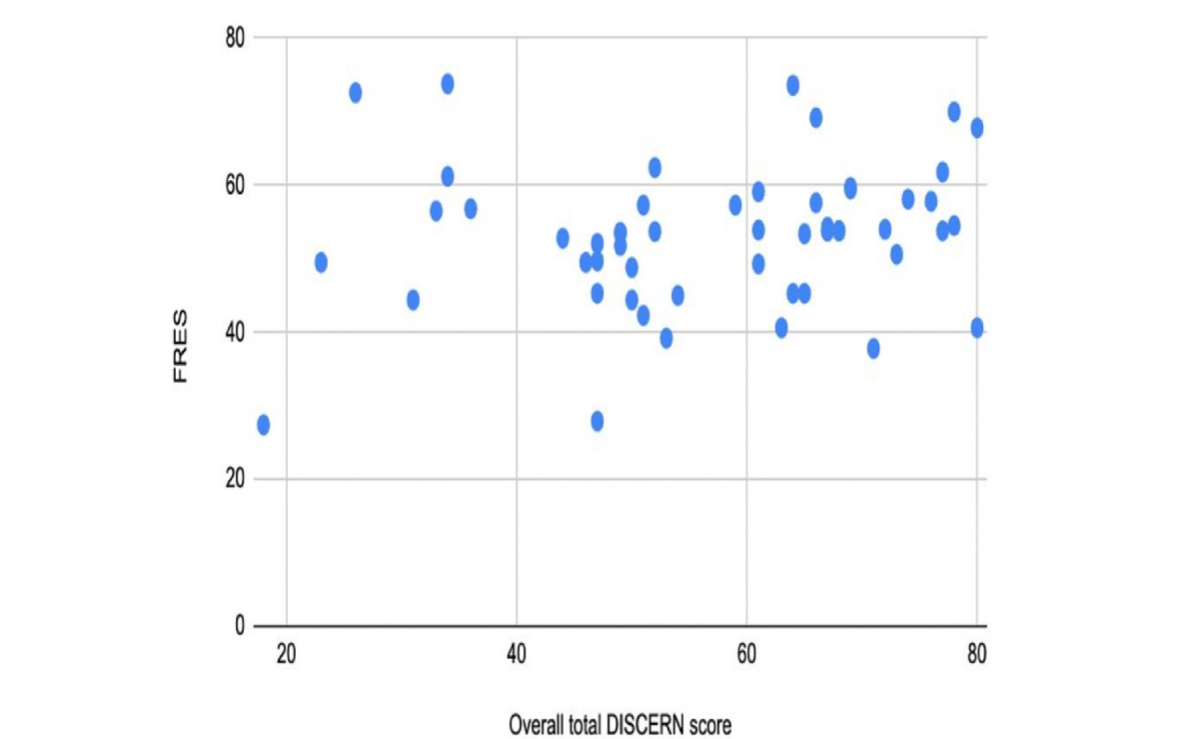
Scatterplot analysis of Flesch Reading Ease score (FRES) versus overall total DISCERN score showing no correlation between content quality and readability.

**Figure 5:**
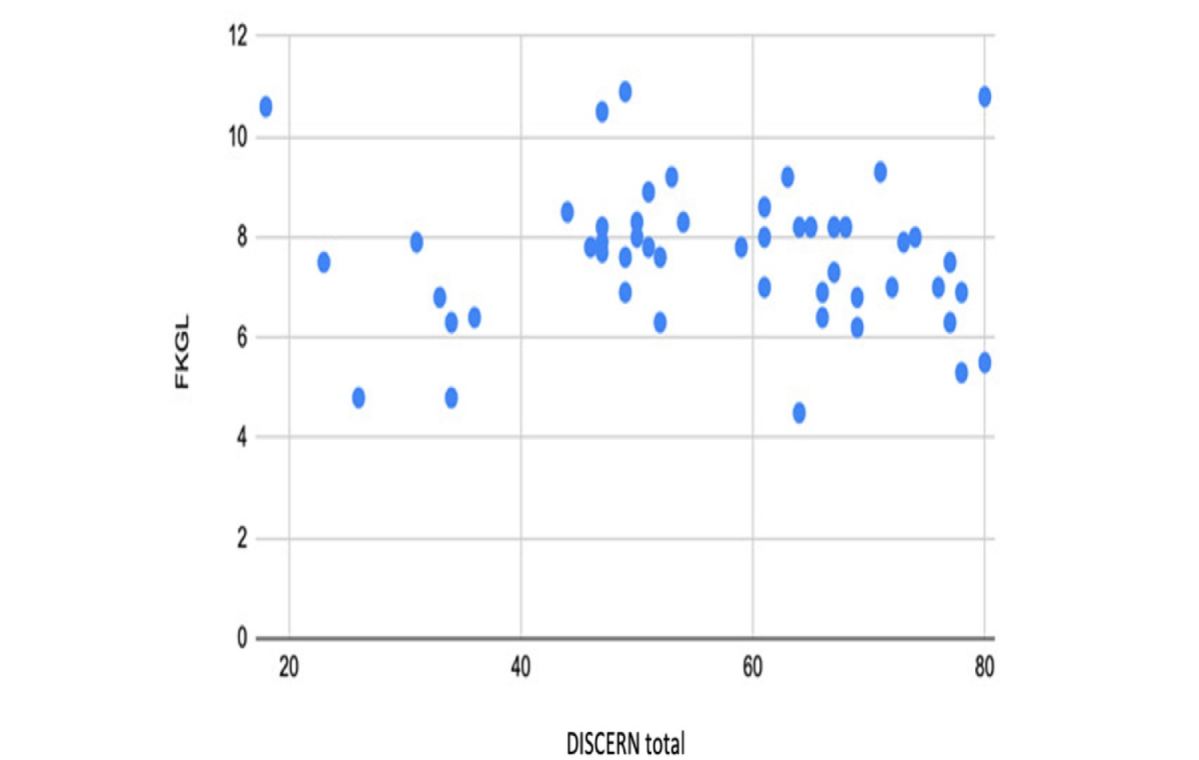
Scatterplot analysis of Flesch-Kincaid grade level (FKGL) versus overall total DISCERN score showing no correlation between content quality and readability.

The overall mean readability scores indicated that the websites as a group were fairly difficult to read. The mean FRES was 53.2 with a range of 27.4 to 73.8. The mean FKGL level across all websites was 7.7 with a range of 4.5 to 10.9. These scores reflected an average reading age of 14.3 years. Only 9% (5/57) of the websites achieved the recommended readability level of sixth grade or lower (Figure 3). Although there was no significant difference in FRES or FKGL between websites of different countries, the FRES of 65 for charity or nongovernmental organization websites was significantly higher than that of hospitals (51/57, 89%; *P*=.02), professional societies (40/57, 70%; *P*=.001), and encyclopedia websites (47/57, 82%; *P*=.04). The FRES of professional societies was also significantly lower than that of news service websites (*P*=.05; Table S3 in Multimedia Appendix 2; Table 9). Similarly, the FKGL of 6 for charity websites was significantly lower than that of academics (8/57, 14%; *P*=.01), hospitals (8/57, 14%; *P*=.01), professional societies (9/57, 16%; *P*<.001), encyclopedias (8/57, 14%; *P*=.03), and general practitioner websites (8/57, 14%; *P*=.01). The FKGL of news services (7/57, 12%; *P*=.008) was also significantly lower than that of professional societies websites. The Pearson correlation scatter plots showed no correlation between the overall DISCERN score and the FRES score and no correlation between the overall DISCERN score and the FKGL (Pearson correlation ranged from −0.2 to +0.2; *P*=.73; Figures 4 and 5). The presence of HONcode certification did not predict a significant difference in any of the readability assessments used and was associated with a lower FRES score.

**Table 9:** Summary of readability assessment using Flesch Reading Ease score (FKGL), Flesch-Kincaid grade level (FRES), Gunning-Fog Score (GFS), and Simple Measure of Gobbledygook (SMOG) indices for websites retrieved between June 2023 and July 2023 containing information about nasopharyngeal carcinoma (N=57).

Source or median readability	FKGL	Reading age (years)	FRES	GFS	SMOG index
Academic center (academic institutions)	53	15	8	8	7
Charity or nongovernmental organization (oversee a broader demographic, such as Red Cross and WHO^a^)	65	13	6	7	6
Encyclopedia	47	15	8	8	7
General practitioner (nonprofit)	54	15	8	10	7
Government or health department	58	15	8	9	7
Hospital (any organization that provides hospital care)	51	15	8	10	7
Industry (for-profit organization within the medical industry, including clinics)	58	15	7	9	7
News service (both primary and secondary news articles that are not written for professionals)	60	14	7	7	5
Professional society (nonprofit groups of health care professionals)	40	16	9	9	7

^a^WHO: World Health Organization.

### Additional Information

In total, 91% (52/57) of the websites mentioned risk factors for NPC, and EBV was the most frequently mentioned (45/57, 79%), followed by a high-salt diet such as consumption of cured meats and fish (40/57, 70%). The third most mentioned risk factors included Human Papilloma Virus, smoking, alcohol, family history, and male sex, all of which were mentioned by 58% (33/57) of the websites. In total, 95% (54/57) of the websites discussed red flag signs and symptoms that help identify NPC. Unexplained neck lump that persisted for >3 weeks was the most common red flag sign mentioned (53/57, 93%), followed by hearing loss (39/57, 68%), unilateral blood-stained rhinorrhea or epistaxis (37/57, 65%), and headaches (34/57, 60%). In total, 82% (47/57) of the websites included investigations and management. The use of imaging modalities such as computerized tomography, magnetic resonance imaging, positron emission tomography, and ultrasound sonography was mentioned the most (44/57, 77%), followed by biopsy of the lesion (42/57, 74%) and nasoendoscopy (35/57, 61%). In terms of management, radiotherapy (51/57, 89%) was mentioned most by the websites, followed by chemotherapy (48/57, 84%) and surgery (46/57, 81%). However, less than half of the websites discussed secondary prevention advice (25/57, 44%), overall mortality (27/57, 47%), and complication rates (23/57, 40%) of NPC.

In total, 61% (35/57) of the websites included posttreatment advice and most mentioned the need for a follow-up appointment, but only 7% (4/57) of the websites educated patients on the signs and symptoms that warrant readmission or reassessment after a treatment procedure. In total, 68% (39/57) of the websites discussed the epidemiology and etiology of NPC, but less than half (28/57, 49%) explained the anatomy and physiology of the head and neck. Finally, only 18% (10/57) of the websites included a section for patient feedback, which is concerning as the written information is targeted for patients mostly. Hence, it is absolutely essential to gather patient feedback for continued development and improvement of a website.

## Discussion

### Principal Findings

Early detection of NPC is vital for achieving significant patient outcomes as treatment response are more favorable in when cancer diagnosed at an early stage. However, this is often hindered by the varied clinical presentation of NPC, nonspecific manifestations such as headaches, and lack of awareness of symptoms, resulting in a high misdiagnosis rate [23,35-40]. Thus, it is vital that information available on the internet is accurate and readable to avoid exacerbating this and increase awareness [6,10,40,41]. To the best of our knowledge, this is the first study to assess the quality and readability of written English language web-based information on NPC using the quality indicators DISCERN, JAMA, and HONcode and the readability tools FRES and FKGL. The aim of this study was also to use that evaluation to provide recommendations for improving website quality and readability.

### Selection of Assessment Tools

DISCERN was selected for this study because of its demonstrated ability to discern high- and low-quality written medical information by both health care professionals and patients [10,42-44]. In addition, in studies conducted by McCool et al [45], DISCERN demonstrated higher interreliability and intrareliability, better agreement, and more precise judgment than Ensuring Quality Information for Patients [45,46]. Furthermore, unlike the Patient Education Materials Assessment Tool tools, which can be used for audio-visual materials, DISCERN was developed to evaluate written information specifically, thus demonstrating its suitability for this study [47-49]. As the use of multiple quality assessment tools improves accuracy and provides a more holistic overview, JAMA, an accountability indicator based on 4 benchmarks, was also selected [3,10].

The selection of readability tools often depends on the goals and field of the study. Although many studies, such as those conducted by LeBrun et al [50], report minimal differences between assessment tools, there are instances where this is not the case [51]. For instance, Wang et al [47] highlighted a significant variance between readability assessment tools. As such, the use of multiple well-validated readability scores is often implemented. FRES and FKG were selected for these studies as they are widely used in education and research settings and are therefore easily interpretable, allowing for broader comparability and consistency [31].

### Quality Assessment

According to the JAMA benchmarks, the website content ranged from poor to moderate quality. The overall JAMA score for all websites assessed was 2.8, with 60% (34/57) of the websites achieving a JAMA score of >2. No website met all the JAMA criteria, which is a recurrent theme in many studies [10,52-55]. However, according to DISCERN scores, the quality of most websites ranged from excellent to fair. The overall DISCERN score for all websites evaluated was 57.6, which is considered “good” according to the criteria, with 28% (16/57) of websites achieving a score of ≥68, which is considered “excellent.” Furthermore, although no statistical differences between websites were found in the quality sections of the DISCERN tool, significant differences in reliability were observed, with hospitals scoring the lowest in this section. JAMA enables quick assessment; however, because minimal guidance is provided for using the tool, the results may be influenced by the user [52,56]. Furthermore, Bharmal and Johal [52] suggested that as JAMA uses binary yes or no questions rather than a scale, it may judge websites more harshly than other tools. This is exemplified by literature, as websites across many disciplines, such as dental care, pediatric care, and oncology, tend to score lower on JAMA benchmarks than other tools [10,52-55,57,58]. In addition, the relevance of JAMA criteria to some websites has been questioned by some researchers. For example, Mac et al [59] reported a limitation in the relevance of JAMA to consumer-focused websites. This may explain why within this study only 46% (26/57) of the websites scored on the “authorship” benchmark and 54% (31/57) of the websites scored on the “attribution” benchmark.

Websites created in the United Kingdom (21/57, 37%) had the highest median total DISCERN score of 67, whereas websites originating from China (1/57, 2%) scored the lowest of 31. A significant number of quality assessment tools are created using English, which may limit their applicability in other countries [48]. This may be remedied by translating and adapting quality assessment tools in other languages, as demonstrated by Shan et al [48] and Logullo et al [60].

### Health Care Websites

Health care websites, such as hospital or general practice websites, performed poorly in both the JAMA and DISCERN quality assessments. Hospital websites scored the lowest according to JAMA benchmarks compared with news, charity nongovernmental, or industry websites. In addition, general practitioner websites failed to score within the “authorship” and “attribution” benchmarks of the JAMA criteria. Similarly, the lowest overall DISCERN scores were obtained for websites affiliated with hospitals, with an overall score of 36.5. Although this is concerning, as health care websites are often the most accessible and trusted by the public and should contain readable, reliable, and accurate information, these findings are consistent with literature. Kuter et al [57] similarly reported that hospital websites did not meet all the JAMA criteria, particularly the attribution, disclosure, and currency criteria. “Inaccurate and incomplete” information in hospital information on the internet has also been reported by Goodman et al [61] and Yee [62]. Furthermore, while developing the STaRNet Website Assessment Tool, Howitt et al [63] reported that the information on general practitioner websites was significantly lacking in quality. Studies that evaluate the lower quality of information on health care websites are minimal; thus, it is evident that more research is required to improve this [63].

### Health on the Net Foundation

Only 1 website [64], which focused on the prognosis of NPC, was HONcode certified. Similarly, Doubleday et al [65] found that a minority of websites for thyroid cancer (40.9%) were HONcode certified. However, that website only achieved a JAMA score of 3 and a DISCERN score of 50, which was lower than the mean. This suggests that HONcode may not be a sufficient means of assessing the quality of web-based information. The HONcode was discontinued in December 2022, and its limitations have been reported in literature [66-68]. Eysenbach [69] reported a potential misunderstanding of the Hon-logo by the general public as an award rather than a voluntary certification, implying that the contents of HONcode-accredited websites are reliable and trustworthy [10,55]. Thus, websites can be HONcode accredited while not meeting all the required principles [51]. Thus, given the significant limitations, HONcode may be more useful when used in combination with other indicators [51,56].

### Readability Assessment

Overall, the websites were fairly difficult to read, as indicated by the readability scores. The overall mean FRES (53.2) and the mean KFGL score (7.7) both indicated a reading level of 14.3 years, significantly exceeding the recommendations of the American Medical Association and the National Institutes of Health [4,16]. Only 9% (5/57) of the websites achieved the recommended sixth grade or lower readability level. This is similar to observations by McKearney and McKearney [70] and Grose et al [71], who both reported an average reading level of 10th grade on websites discussing ear tubes and neck dissections, respectively. Websites with information with a reading grade higher than the recommended level were also reported by Raja and Fitzpatrick [29], De La Chapa et al [72], Crabtree and Lee [3], and Kim et al [73], thus demonstrating the poor readability of health care websites in general.

Similar to the quality assessments, charity and nongovernmental websites achieved the highest FRES score (n=65) and were therefore easier to read than other websites, whereas professional society websites achieved the lowest readability scores. Studies conducted by Charow et al [74] reported better readability with professional society websites compared with those affiliated with charities and nongovernmental organizations, whereas Bould and Forshaw [75] found varied readability within charity and nongovernmental websites. Thus, the observations in this study may not be applicable to all health care fields.

No correlation was found between the overall DISCERN score and FRES scores, or between DISCERN and FKG, as determined by the Pearson correlation analysis. This is consistent with the studies conducted by Lee et al [76] and Hong et al [77], who reported no correlation between readability and quality scores. However, this differs significantly from the study conducted by Grose et al [71] and Raja and Patel [78], who reported positive correlations between DISCERN, FRES, and FKG, and therefore suggested that higher-quality websites are often more reliable and more likely to be readable. Thus, a more exhaustive investigation of the correlation between FKG, FRES, and DISCERN is required.

### Ear, Nose, and Throat Studies

Our findings are consistent with some ear, nose, and throat studies, as Duymaz et al [79] evaluated the quality of pediatric tracheostomy care information on YouTube and reported low JAMA scores for channels managed by both health care professionals and independent users. Similarly, only a small percentage of thyroid cancer websites assessed by Doubleday et al [65] satisfied the JAMA criteria, whereas the same websites were classified as “fair” when the DISCERN tool was applied. In addition, Eker et al [80] investigated the accuracy, reliability, and understandability of NPC information on YouTube. They reported that videos produced by medical institutions and universities did not have improved accuracy, reliability, or usefulness compared with other groups [80]. This is consistent with the findings of this study, as NPC websites affiliated with academic centers, such as universities or medical schools, had the second lowest DISCERN score of 49, which is considered “fair” according to the criteria. Similar to health care websites, academic websites are expected to have high-quality and reliable information because of their educational nature, and as such, a more intensive investigation is required to elucidate this.

### Alternative Indicators

In addition to the quality and readability scores, the presence of relevant content was assessed. Less than half of the websites evaluated discussed the use of secondary prevention methods (25/57, 44%) or mentioned the overall mortality (27/57, 47%) and complication rates (23/57, 40%). This was concerning as it suggests that risk factors for NPC, such as viral infections or high salt intake, and subsequent complications are not being communicated adequately. In addition, although most websites (39/57, 68%) discussed NPC epidemiology and etiology, less than half of the websites (28/57, 49%) provided adequate explanations of the anatomy and physiology of the head and neck and the nasopharynx, which may hinder patient comprehension. Finally, only 18% (10/57) of the websites included a section for patient feedback, despite the written information being targeted to patients, thus hindering patient-focused development and improvement of websites.

### Limitations

Although the use of interrater reliability aims to reduce subjective bias, the JAMA and DISCERN tools rely heavily on subjective measures of quality, thus preventing the complete elimination of subjective bias. In addition, only 1 “almost perfect” agreement was achieved for DISCERN scores, thus highlighting the need to account for the influence of human bias when interpreting results using both JAMA and DISCERN tools. Only websites created in English were evaluated in this study; however, patients may encounter web-based material in other languages, which would be outside the scope of this study. Thus, the conclusions drawn may not be applicable to non–English-speaking populations and may not represent the information available in other languages. Translation tools may be used to incorporate non-English language websites into future studies. Keyword selection using Google Trends was a significant limitation because the specific number of queries was not disclosed to the public. Therefore, the most frequently used search terms were determined by gauging the relative popularity of individual keywords in comparison with each other. Owing to the dynamic nature of the internet, search engine results, website content, and search trends may change over time. As our study only assessed websites at the time of search, our results are limited to a snapshot of a website’s content and relative popularity. Finally, patient understanding was not evaluated in this study; thus, readability may differ between patients who have experienced symptoms associated with NPC and those who have not.

### Further Recommendations

Further recommendations include using artificial intelligence (AI) systems to generate and validate health content. Although AI may currently be limited, further development and carefully regulated incorporation into health communications may enhance the quality and readability of medical content [81]. Future studies should analyze the extent of the benefits and drawbacks of AI-generated health content. In addition, additional guidance for using tools such as JAMA and DISCERN to generate and assess content should be provided to encourage the creation of accurate and reliable health care information. We also recommend creating community-level initiatives to increase public awareness of NPC symptoms and prevention strategies, such as lifestyle changes or ensuring vaccinations are up-to-date.

### Conclusions

This study provides valuable insights into the quality and readability of NPC websites across many categories. NPC websites exhibited varied quality, with most websites achieving low JAMA scores; however, DISCERN scores indicated information that ranged from “fair” to “excellent” quality. Patients may struggle to read and understand NPC websites due to the level of difficulty comprehending web-based content as it exceeds the recommended US grade level of 6 and the omission of essential information. Surprisingly, health care and academic websites contained information with poorer readability and lower quality content overall. Taken together, this highlights the importance of improving NPC content for health care and educational websites and highlights the need for continued development of quality and readability assessment tools.

## Appendix

Multimedia Appendix 1 List of unique websites evaluated from the 3 search engines (Google, Yahoo, and Bing) for information on nasopharyngeal carcinoma after applying the exclusion criteria.

Multimedia Appendix 2 Overall statistical analysis of Journal of the American Medical Association and DISCERN scores over different website sources.

Multimedia Appendix 3 Number of websites meeting the Journal of the American Medical Association’s category-specific criteria for nasopharyngeal carcinoma.

## Abbreviations

AI: artificial intelligence
EBV: Epstein-Barr virus
FKGL: Flesch-Kincaid grade level
FRES: Flesch Reading Ease score
GFS: Gunning-Fog Score
HONcode: Health on the Net Foundation
JAMA: Journal of the American Medical Association
NPC: nasopharyngeal carcinoma
PRISMA: Preferred Reporting Items for Systematic Reviews and Meta-Analyses
SMOG: Simple Measure of Gobbledygook
