# Supplementary material for: The Readability and Quality of Web-Based Patient Information on Nasopharyngeal Carcinoma: Quantitative Content Analysis
Source: JMIR Form Res. 2023 Nov 27;7:e47762. doi: 10.2196/47762 (PMC10714271; doi:10.2196/47762)
Supplement: Multimedia Appendix 1 [file formative_v7i1e47762_app1.docx]

**Table S1.** List of unique websites evaluated from the 3 search engines (Google, Yahoo, and Bing) for information on nasopharyngeal carcinoma after applying the exclusion criteria.

| Website domain (N=57) |
| --- |
| <https://www.healthdirect.gov.au/throat-cancer> |
| <https://www.betterhealth.vic.gov.au/health/conditionsandtreatments/throat-cancer> |
| <https://www.cancer.org.au/cancer-information/types-of-cancer/throat-cancer> |
| <http://www.bccancer.bc.ca/health-info/types-of-cancer/head-neck/pharynx#Diagnosis--&--staging> |
| <https://www.shine.cn/feature/wellness/2302146206/> |
| <https://www.macmillan.org.uk/cancer-information-and-support/head-and-neck-cancer> |
| <https://www.macmillan.org.uk/cancer-information-and-support/head-and-neck-cancer/nasopharyngeal-cancer> |
| <https://www.macmillan.org.uk/cancer-information-and-support/head-and-neck-cancer/throat-cancer> |
| <https://www.medicalnewstoday.com/articles/312087#summary> |
| <https://www.cancerresearchuk.org/about-cancer/nasopharyngeal-cancer> |
| <https://www.cancerresearchuk.org/about-cancer/head-neck-cancer/throat> |
| <https://111.wales.nhs.uk/encyclopaedia/c/article/cancer,nasopharyngeal/> |
| <https://www.nhsinform.scot/illnesses-and-conditions/cancer/cancer-types-in-adults/nasopharyngeal-cancer> |
| <https://www.madeiramedical.nhs.uk/conditions/nasopharyngeal-cancer/> |
| <https://www.stoneleighsurgery.nhs.uk/conditions/nasopharyngeal-cancer/> |
| <https://www.woodcockroadsurgery.nhs.uk/conditions/nasopharyngeal-cancer/> |
| <https://www.aspenmedicalpractice.nhs.uk/conditions/nasopharyngeal-cancer/> |
| <https://www.elthampalacesurgery.nhs.uk/conditions/nasopharyngeal-cancer/> |
| <https://www.haslemerehc.nhs.uk/conditions/nasopharyngeal-cancer/> |
| <https://www.stlukessurgeryguildford.nhs.uk/conditions/nasopharyngeal-cancer/> |
| <https://www.nhs.uk/conditions/head-and-neck-cancer/> |
| <https://www.nhs.uk/conditions/nasopharyngeal-cancer/> |
| <https://www.christie.nhs.uk/patients-and-visitors/your-treatment-and-care/types-of-cancer/nasopharyngeal-cancer> |
| <https://www.nidirect.gov.uk/conditions/nasopharyngeal-cancer> |
| <https://www.uclh.nhs.uk/our-services/find-service/cancer-services/head-and-neck-cancer/types-head-and-neck-cancer/nasopharyngeal-cancer> |
| <https://www.uclh.nhs.uk/our-services/find-service/cancer-services/head-and-neck-cancer/types-head-and-neck-cancer/throat-pharynx-cancer> |
| <https://www.mayoclinic.org/diseases-conditions/throat-cancer/symptoms-causes/syc-20366462> |
| <https://www.mayoclinic.org/diseases-conditions/nasopharyngeal-carcinoma/symptoms-causes/syc-20375529> |
| <https://my.clevelandclinic.org/health/diseases/21661-nasopharyngeal-cancer> |
| <https://www.mayoclinic.org/diseases-conditions/nasopharyngeal-carcinoma/symptoms-causes/syc-20375529> |
| <https://my.clevelandclinic.org/health/diseases/23136-throat-cancer> |
| <https://www.cancer.org/cancer/types/nasopharyngeal-cancer/about/what-is-nasopharyngeal-cancer.html> |
| <https://www.asbestos.com/cancer/pharyngeal/> |
| <https://www.healthline.com/health/cancer-throat-or-larynx> |
| <https://www.healthline.com/health/cancer/nasopharyngeal-cancer> |
| <https://www.medicinenet.com/is_nasopharyngeal_cancer_curable/article.htm> |
| <https://www.webmd.com/oral-health/guide/throat-cancer-symptoms-treatments> |
| <https://www.webmd.com/cancer/nasopharyngeal-cancer> |
| <https://emedicine.medscape.com/article/988165-overview> |
| <https://www.emedicinehealth.com/what_is_survival_rate_of_nasopharyngeal_carcinoma/article_em.htm> |
| <https://www.cancer.net/cancer-types/nasopharyngeal-cancer/types-treatment> |
| <https://www.epainassist.com/cancer/what-is-the-prognosis-for-nasopharyngeal-cancer> |
| <https://blog.dana-farber.org/insight/2021/06/what-is-nasopharyngeal-cancer/> |
| <https://www.cancercenter.com/cancer-types/throat-cancer/types/pharyngeal-cancer> |
| <https://facty.com/conditions/cancer/10-symptoms-of-nasopharyngeal-cancer/1/> |
| <https://en.wikipedia.org/wiki/Head_and_neck_cancer> |
| <https://en.wikipedia.org/wiki/Nasopharyngeal_carcinoma> |
| <https://www.cancer.gov/types/head-and-neck/head-neck-fact-sheet> |
| <https://www.cancer.gov/types/head-and-neck/head-neck-fact-sheet> |
| <https://www.hopkinsmedicine.org/health/conditions-and-diseases/nasopharyngeal-carcinoma> |
| <https://www.cancer.gov/types/head-and-neck/patient/adult/nasopharyngeal-treatment-pdq> |
| <https://www.cedars-sinai.org/health-library/diseases-and-conditions/n/nasopharyngeal-cancer.html> |
| <https://www.cedars-sinai.org/health-library/diseases-and-conditions/p/pharyngeal-cancer.html> |
| <https://www.aurorahealthcare.org/services/cancer/head-neck-cancer/pharyngeal-cancer> |
| <https://www.mskcc.org/cancer-care/types/throat/throat-cancer-symptoms> |
| <https://www.mdanderson.org/cancer-types/throat-cancer.html> |
| <https://www.yalemedicine.org/conditions/throat-cancer> |
