# Supplementary material for: The Readability and Quality of Web-Based Patient Information on Nasopharyngeal Carcinoma: Quantitative Content Analysis
Source: JMIR Form Res. 2023 Nov 27;7:e47762. doi: 10.2196/47762 (PMC10714271; doi:10.2196/47762)

Supplementary tables

|  | Industry | News Services | Charity/ Non-governmental organisations | Professional society | Encyclopaedia | Government/ Health Department | Academic centre or institution | General Practitioner | Hospital |
| --- | --- | --- | --- | --- | --- | --- | --- | --- | --- |
| Industry | NA | 0.2  (p =1) | 0.3  (p = 1) | 0.5  (p = 0.998) | 0.8  (p = 0.669) | 1.5  (p = 0.345) | 1.6  (p = 0.218) | 2.0  (p = 0.059) | **2.2**  **(p = 0.037)** |
| News Services | -0.2  (p = 1) | NA | 0.1  (p = 1) | 0.3  (p = 1) | 0.6  (p = 0.82) | 1.3  (p = 0.47) | 1.4  (p = 0.30) | 1.8  (p = 0.078) | **2.0**  **(p = 0.048)** |
| Charity/ Non-governmental organisations | -0.3  (p = 1) | -0.1  (p = 1) | NA | 0.2  (p = 1) | 0.5  (p = 0.801) | 1.2  (p = 0.41) | 1.3  (p = 0.203) | **1.7**  **(p = 0.045)** | **1.9**  **(p = 0.027)** |
| Professional society | -0.5  (p = 0.998) | -0.3  (p = 1) | -0.2  (p = 1) | NA | 0.3  (p = 0.967) | 1.0  (p = 0.742) | 1.1  (p = 0.592) | 1.5  (p = 0.202) | 1.7  (p = 0.131) |
| Encyclopaedia | -0.8  (p = 0.669) | -0.6  (p = 0.818) | -0.5  (p = 0.801) | -0.3  (p = 0.967) | NA | 0.7  (p = 0.998) | 0.8  (p = 0.995) | 1.2  (p = 0.706) | 1.4  (p = 0.537) |
| Government/ Health Department | -1.5  (p = 0.345) | -1.3  (p = 0.468) | -1.2  (p = 0.41) | -1.0  (p = 0.742) | -0.7  (p = 0.998) | NA | 0.1  (p = 1) | 0.5  (p = 0.985) | 0.7  (p = 0.936) |
| Academic centre or institution | -1.6  (p = 0.218) | -1.4  (p = 0.296) | -1.3  (p = 0.203) | -1.1  (p = 0.592) | -0.8  (p = 0.995) | -0.1  (p = 1) | NA | 0.4  (p = 0.956) | 0.6  (p = 0.85) |
| General Practitioner | -2  (p = 0.059) | -1.8  (p = 0.078) | **-1.7**  **(p = 0.045)** | -1.5  (p = 0.202) | -1.2  (p = 0.706) | -0.5  (p = 0.985) | -0.4  (p = 0.956) | NA | 0.2  (p = 1) |
| Hospital | **-2.2**  **(p = 0.037)** | **-2.0**  **(p = 0.048)** | **-1.9**  **(p = 0.027)** | -1.7  (p = 0.131) | -1.4  (p = 0.537) | -0.7  (p = 0.936) | -0.6  (p = 0.85) | -0.2  (p = 1) | NA |

**Supplementary Table A** Mean JAMA score comparison between different website types for nasopharyngeal cancer. NA = Not Applicable. Figures that are bolded indicate statistically significant results (P <.05)

**Supplementary Table B** Median total DISCERN score comparison between different website types for nasopharyngeal cancer. NA = Not Applicable. Figures that are bolded indicate statistically significant results (P < .05)

|  | Industry | News Services | Charity/ Non-governmental organisations | Professional society | Encyclopaedia | Government/ Health Department | Academic centre or institution | General Practitioner | Hospital |
| --- | --- | --- | --- | --- | --- | --- | --- | --- | --- |
| Industry | NA | -0.5  (p =1) | -11.5  (p = 1) | 3.5  (p = 1) | 9.0  (p = 0.989) | 10.5  (p = 1) | 17.0  (p = 0.957) | -1.0  (p = 1) | 29.5  (p = 0.288) |
| News Services | 0.5  (p = 1) | NA | -11.0  (p = 1) | 4.0  (p = 0.999) | 9.5  (p = 1) | 11.0  (p = 1) | 17.5  (p = 1) | -0.5  (p = 0.982) | 30.0  (p = 0.567) |
| Charity/ Non-governmental organisations | 11.5  (p = 1) | 11.0  (p = 1) | NA | 15.0  (p = 1) | 20.5  (p = 0.975) | 22.0  (p = 1) | 28.5  (p = 0.885) | 10.5  (p = 1) | 41.0  (p = 0.118) |
| Professional society | -3.5  (p = 1) | -4.0  (p = 0.999) | -15.0  (p = 1) | NA | 5.5  (p = 0.981) | 7.0  (p = 1) | 13.5  (p = 0.926) | -4.5  (p = 1) | 26.0  (p = 0.193) |
| Encyclopaedia | -9.0  (p = 0.989) | -9.5  (p = 1) | -20.5  (p = 0.975) | -5.5  (p = 0.981)_ | NA | 1.5  (p = 1) | 8.0  (p = 1) | -10.0  (p = 0.807) | 20.5  (p = 0.604) |
| Government/ Health Department | -10.5  (p = 1) | -11.0  (p = 1) | -22.0  (p = 1) | -7.0  (p = 1) | -1.5  (p = 1) | NA | 6.5  (p = 0.995) | -11.5  (p = 0.988) | 19.0  (p = 0.307) |
| Academic centre or institution | -17.0  (p = 0.957) | -17.5  (p = 1) | -28.5  (p = 0.885) | -13.5  (p = 0.926) | -8.0  (p = 1) | -6.5  (p = 0.995) | NA | -18.0  (p = 0.526) | 12.5  (p = 0.554) |
| General Practitioner | 1.0  (p = 1) | 0.5  (p = 0.982) | -10.5  (p = 1) | 4.5  (p = 1) | 10.0  (p = 0.807) | 11.5  (p = 0.988) | 18.0  (p = 0.526) | NA | **30.5**  **(p = 0.03)** |
| Hospital | -29.5  (p = 0.288) | -30.0  (p = 0.567) | -41.0  (p = 0.118) | -26.0  (p = 0.193) | -20.5  (p = 0.604) | -19.0  (p = 0.307) | -12.5  (p = 0.554) | -**30.5**  **(p = 0.03)** | NA |

**Supplementary Table C** Median DISCERN reliability score comparison between different website types for nasopharyngeal cancer. NA = Not Applicable. Figures that are bolded indicate statistically significant results (P < .05).

|  | Industry | News Services | Charity/ Non-governmental organisations | Professional society | Encyclopaedia | Government/ Health Department | Academic centre or institution | General Practitioner | Hospital |
| --- | --- | --- | --- | --- | --- | --- | --- | --- | --- |
| Industry | NA | -2.0  (p = 0.993) | -4.0  (p = 1) | -1.5  (p = 1) | 0.5  (p = 0.987) | 2.5  (p = 0.997) | 6.0  (p = 0.692) | 0.0  (p = 1) | **15.5**  **(p = 0.046)** |
| News Services | 2.0  (p = 0.993) | NA | -2.0  (p = 0.993) | 0.5  (p = 0.996) | 2.5  (p = 1) | 4.5  (p = 1) | 8.0  (p = 0.996) | 2.0  (p = 0.995) | 17.5  (p = 0.231) |
| Charity/ Non-governmental organisations | 4.0  (p = 1) | 2.0  (p = 0.993) | NA | 2.5  (p = 1) | 4.5  (p = 0.982) | 6.5  (p = 0.997) | 10.0  (p = 0.476) | 4.0  (p = 1) | **19.5**  **(p = 0.01)** |
| Professional society | 1.5  (p = 1) | -0.5  (p = 0.996) | -2.5  (p = 1) | NA | 2.0  (p = 0.99) | 4.0  (p = 0.999) | 7.5  (p = 0.654) | 1.5  (p = 1) | **17.0**  **(p = 0.029)** |
| Encyclopaedia | -0.5  (p = 0.987) | -2.5  (p = 1) | -4.5  (p = 0.982) | -2.0  (p = 0.99) | NA | 2.0  (p = 1) | 5.5  (p = 0.984) | -0.5  (p = 0.985) | 15.0  (p = 0.098) |
| Government/ Health Department | -2.5  (p = 0.997) | -4.5  (p = 1) | -6.5  (p = 0.997) | -4.0  (p = 0.999) | -2.0  (p = 1) | NA | 3.5  (p = 0.949) | -2.5  (p = 0.998) | 18.5  (p = 0.075) |
| Academic centre or institution | -6.0  (p = 0.692) | -8.0  (p = 0.996) | -10.0  (p = 0.476) | -7.5  (p = 0.654) | -5.5  (p = 0.984) | -3.5  (p = 0.949) | NA | -6.0  (p = 0.453) | 9.5  (p = 0.291) |
| General Practitioner | 0.0  (p = 1) | -2.0  (p = 0.995) | -4.0  (p = 1) | -1.5  (p = 1) | 0.5  (p = 0.985) | 2.5  (p = 0.998) | 6.0  (p = 0.453) | NA | **15.5**  **(p = 0.008)** |
| Hospital | **-15.5**  **(p = 0.046)** | -17.5  (p = 0.231) | **-19.5**  **(p = 0.01)** | **-17.0**  **(p = 0.029)** | -15.0  (p = 0.098) | -13.0  (p = 0.075) | -9.5  (p = 0.291) | **-15.5**  **(p = 0.008)** | NA |

**Supplementary Table D** Median FRES comparison between different website types for nasopharyngeal cancer.

NA = Not Applicable. Figures that are bolded indicate statistically significant results (P<.05).

|  | Industry | News Services | Charity/ Non-governmental organisations | Professional society | Encyclopaedia | Government/ Health Department | Academic centre or institution | General Practitioner | Hospital |
| --- | --- | --- | --- | --- | --- | --- | --- | --- | --- |
| Industry | NA | -2.0  (p = 1) | -7.0  (p = 0.94) | 18.0  (p = 0.19) | 11.0  (p = 0.871) | 0.0  (p = 1) | 5.0  (p = 0.987) | 4.0  (p = 1) | 7.0  (p = 0.757) |
| News Services | 2.0  (p = 1) | NA | -5.0  (p = 0.99) | **20.0**  **(p = 0.046)** | 13.0  (p = 0.514) | 2.0  (p = 1) | 7.0  (p = 0.806) | 6.0  (p = 0.973) | 9.0  (p = 0.374) |
| Charity/ Non-governmental organisations | 7.0  (p = 0.94) | 5.0  (p = 0.99) | NA | **25.0**  **(p = 0.001)** | **18.0**  **(p = 0.035)** | 7.0  (p = 0.883) | 12.0  (p = 0.077) | 11.0  (p = 0.36) | **14.0**  **(p = 0.02)** |
| Professional society | -18.0  (p = 0.19) | **-20.0**  **(p = 0.046)** | **-25.0**  **(p = 0.001)** | NA | -7.0  (p = 0.778) | -18.0  (p = 0.051) | -13.0  (p = 0.259) | -14.0  (p = 0.211) | -11.0  (p = 0.924) |
| Encyclopaedia | -11.0  (p = 0.871) | -13.0  (p = 0.514) | **-18.0**  **(p = 0.035)** | 7.0  (p = 0.778) | NA | -11.0  (p = 0.608) | -6.0  (p = 0.995) | -7.0  (p = 0.966) | -4.0  (p = 1) |
| Government/ Health Department | 0.0  (p = 1) | -2.0  (p = 1) | -7.0  (p = 0.883) | 18.0  (p = 0.051) | 11.0  (p = 0.608) | NA | 5.0  (p = 0.897) | 4.0  (p = 0.995) | 7.0  (p = 0.446) |
| Academic centre or institution | -5.0  (p = 0.987) | -7.0  (p = 0.806) | -12.0  (p = 0.077) | 13.0  (p = 0.259) | 6.0  (p = 0.995) | -5.0  (p = 0.897) | NA | -1.0  (p = 1) | 2.0  (p = 0.959) |
| General Practitioner | -4.0  (p = 1) | -6.0  (p = 0.973) | -11.0  (p = 0.36) | 14.0  (p = 0.211) | 7.0  (p = 0.966) | -4.0  (p = 0.995) | 1.0  (p = 1) | NA | 3.0  (p = 0.885) |
| Hospital | -7.0  (p = 0.757) | -9.0  (p = 0.374 | **-14.0**  **(p = 0.02)** | 11.0  (p = 0.924) | 4.0  (p = 1) | -7.0  (p = 0.446) | -2.0  (p = 0.959) | -3.0  (p = 0.885) | NA |

Figure 2 Number of websites meeting the Journal of the American Medical Association JAMA’s category-specific criteria for nasopharyngeal carcinoma.


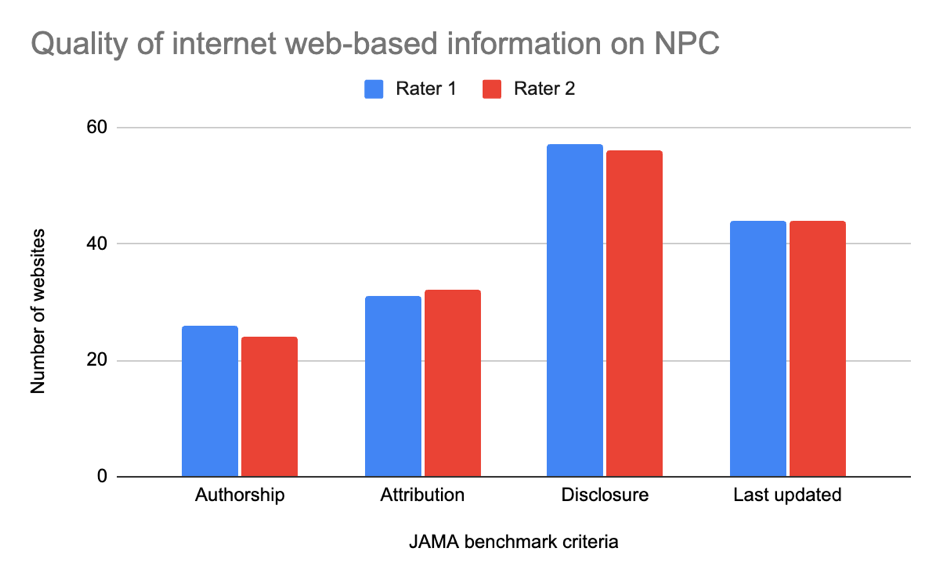


Figure 3. Overall DISCERN score for nasopharyngeal cancer.


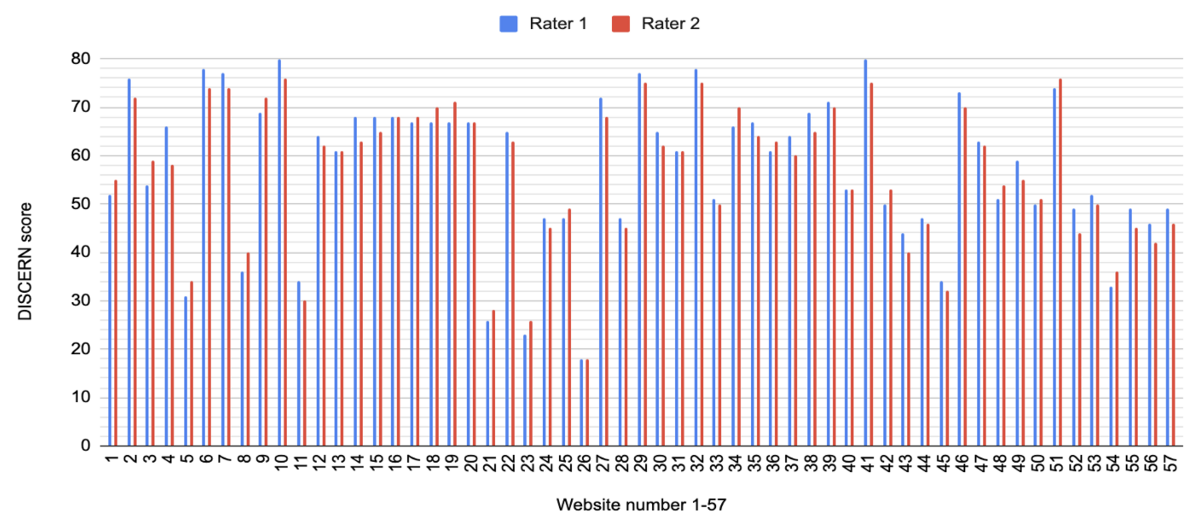

Supplement: Multimedia Appendix 2 [file formative_v7i1e47762_app2.docx]
