# Supplementary figures and images for: The Readability and Quality of Web-Based Patient Information on Nasopharyngeal Carcinoma: Quantitative Content Analysis
Source: JMIR Form Res. 2023 Nov 27;7:e47762. doi: 10.2196/47762 (PMC10714271; doi:10.2196/47762)

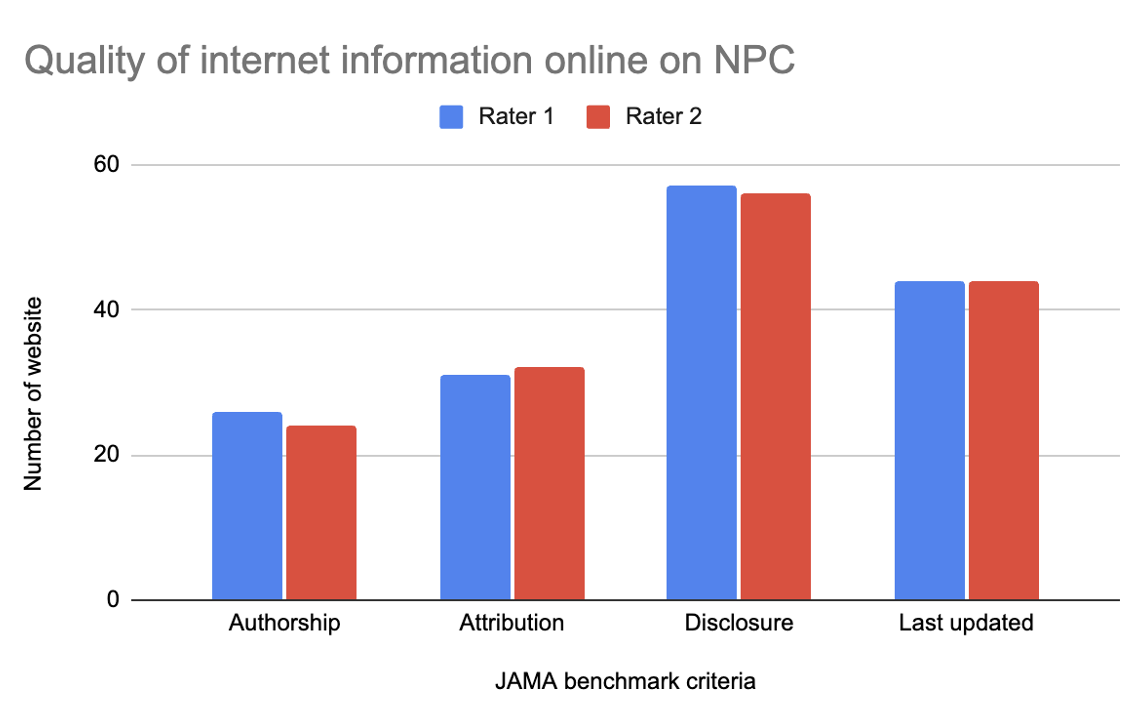

Supplement: Multimedia Appendix 3 [file formative_v7i1e47762_app3.png]
